# Fine-scale spatial variation in fitness, inbreeding, and inbreeding depression in a wild ungulate

**DOI:** 10.1093/evlett/qrae073

**Published:** 2025-01-08

**Authors:** Anna M Hewett, Susan E Johnston, Gregory F Albery, Alison Morris, Sean J Morris, Josephine M Pemberton

**Affiliations:** Institute of Ecology and Evolution, School of Biological Sciences, University of Edinburgh, Edinburgh, United Kingdom; Institute of Ecology and Evolution, School of Biological Sciences, University of Edinburgh, Edinburgh, United Kingdom; School of Natural Sciences, Trinity College Dublin, Dublin, Ireland; Institute of Ecology and Evolution, School of Biological Sciences, University of Edinburgh, Edinburgh, United Kingdom; Institute of Ecology and Evolution, School of Biological Sciences, University of Edinburgh, Edinburgh, United Kingdom; Institute of Ecology and Evolution, School of Biological Sciences, University of Edinburgh, Edinburgh, United Kingdom

**Keywords:** inbreeding, inbreeding depression, spatial variation, inbreeding depression-by-environment interaction

## Abstract

Environmental stress can exacerbate inbreeding depression by amplifying differences between inbred and outbred individuals. In wild populations, where the environment is often unpredictable and stress can be highly detrimental, the interplay between inbreeding depression and environmental variation is potentially important. Here, we investigate variation in inbreeding level, fitness and strength of inbreeding depression across a fine-scale geographic area (~12 km^2^) in an individually monitored population of red deer (*Cervus elaphus*). We show that northern regions of the study area have lower birth weights, lower juvenile survival rates, and higher inbreeding coefficients. Such fine-scale differences in inbreeding coefficients could be caused by the mating system of red deer combined with female density variation. We then tested for an inbreeding depression-by-environment interaction (ID × E) in birth weight and juvenile survival, by fitting an interaction term between the inbreeding coefficient and geographic location. We find that inbreeding depression in juvenile survival is stronger in the harsher northern regions, indicating the presence of ID × E. We also highlight that the ability to infer ID *×* E is affected by the variation in inbreeding within each geographic region. Therefore, for future studies on ID *×* E in wild populations, we recommend first assessing whether inbreeding and traits vary spatially or temporally. Overall, this is one of only a handful of studies to find evidence for ID *×* E in a wild population—despite its prevalence in experimental systems—likely due to intense data demands or insufficient variation in environmental stress or inbreeding coefficients.

## Introduction

Both inbreeding and environmental stress can have major effects on an individual’s fitness. Inbred individuals commonly have lower fitness compared to outbred ones (i.e., inbreeding depression), and living in stressful environments also lowers individual fitness relative to benign environments. It is therefore understandable that exposure to stressful environments can exacerbate the fitness costs associated with inbred individuals (Keller & Waller, 2002; Reed et al., 2012). Environmental stress from climate change, introduced species, or pollution may then have highly detrimental effects on more inbred populations, possibly even leading to extinction (Liao & Reed, 2009). A classic example comes from a population of song sparrows (*Melospiza melodia*), where strong inbreeding depression was revealed by a severe winter storm (Keller et al., 1994). However, further studies are needed to fully understand the interaction between inbreeding depression and the environment (ID × E) in the wild (Keller et al., 2002; Niskanen et al., 2020; Reed et al., 2012).

ID × E is well-documented in experimental systems (Fox & Reed, (2011) and reviewed in Armbruster & Reed (2005)), but it has been suggested that experimental studies impose higher levels of inbreeding and environmental stressors than would be experienced in the wild, enhancing this interaction (Cheptou & Donohue, 2011; Pemberton et al., 2017). In theory, the impact of ID × E in the wild may be even more severe than in the lab, as wild populations may be unable to recover, leading to a heightened risk of extinction (Liao & Reed, 2009; Reed et al., 2012). However, estimating ID × E interactions in a natural population is difficult (Marr et al., 2006; Pemberton et al., 2017). Such a study requires individual fitness data and inbreeding coefficients (either estimated through a pedigree or genomic data), as well as quantifiable environmental stress for large samples of individuals. Consequently, relatively few studies have tested this interaction in wild animal populations, and even fewer detect a significant interaction (reviewed by Pemberton et al. (2017)). One example of ID × E in situ was shown in a population of cactus finch (*Geospiza scandens*), where inbreeding depression in survival was detected only when rainfall was low, and was more severe at higher population density (Keller et al., 2002). A small number of other studies detect ID × E in situ in birds (de Boer et al., 2015; Marr et al., 2006; Richardson et al., 2004; Szulkin & Sheldon, 2007) and mammals (Coltman et al., 1999; Nielsen et al., 2012). However, as these studies quantified inbreeding via potentially inaccurate pedigrees or microsatellite heterozygosity, it is useful to re-examine the topic using the more accurate genomic inbreeding estimators now available.

**Figure 1.**
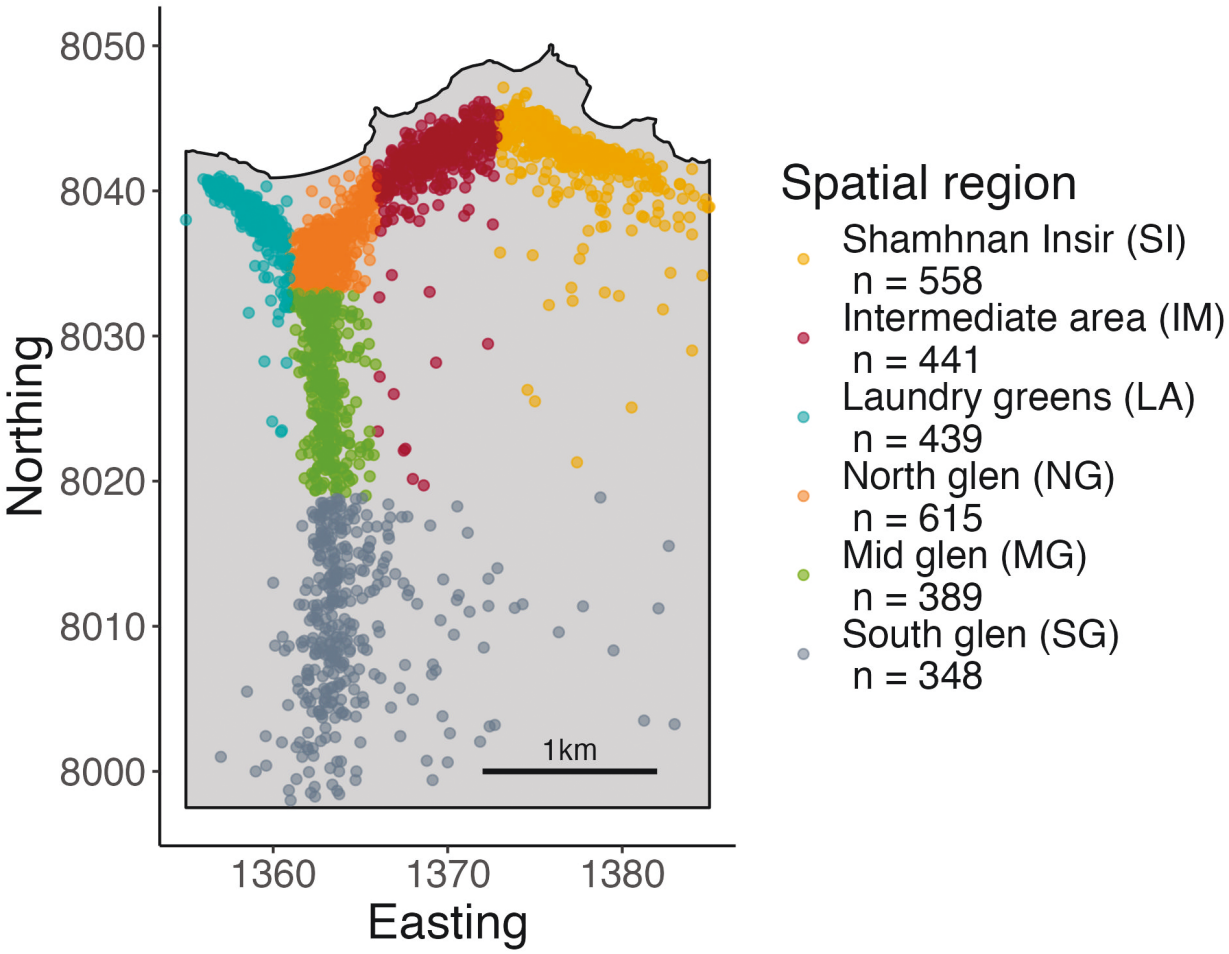
Map of the Isle of Rum deer study area showing the mean northing and easting locations of the mothers of genotyped calves living within the six spatial regions in the first year of the calf’s life. Sample sizes are the number of calves analyzed. Easting and northing are in units of 100 m grid squares, where 10 units correspond to 1 km. The *Y*-shaped distribution of individuals is a result of the distribution of favored grazing, which is along the coast at the north and north–south along the river banks.

The red deer (*Cervus elaphus*) population inhabiting the north block of the Isle of Rum, Scotland, is a good candidate population in which to study ID × E interactions in situ. This unmanaged population experiences inbreeding depression (Hewett et al., 2024; Huisman et al., 2016; Walling et al., 2011) and there is extensive data available on variable environmental stressors, including spatial habitat quality as well as temporal variation. Indeed, using the pedigree information then available, Walling et al. (2011) investigated the impact of temporal variation in spring temperature and winter and autumn rainfall on inbreeding depression in survival to one year. However, they found no evidence of a significant interaction. Here, we investigate the potential for ID × E interactions in space, rather than time. Within the study area boundary, there are six broadly classified spatial subdivisions within which individual deer range (Figure 1) (Gauzere et al., 2022; Huisman et al., 2016). Past studies show that deer residents in the north-east have lower birth weights and survival compared to the rest of the study area, in part due to the higher population density in the north (Coulson et al., 1997; Guinness et al., 1978a, 1978b, see Figure 1 and Supplementary Figure 1). In conjunction with improved precision of individual inbreeding coefficients from genomic data, these previous associations between environmental variables and calf mortality provide an appropriate basis for investigating ID × E across the six spatial regions of the study area. Our aims were threefold: (1) to reanalyze the spatial distribution of two fitness traits (birth weight and juvenile survival) using more years of data; firstly by treating location as a discrete factor using updated subdivisions, and then by accounting for spatial autocorrelation using individual centroids; (2) investigate the spatial distribution of genomic inbreeding coefficients; and (3) assess ID × E as differences in the strength of inbreeding depression across spatial regions.

## Methods

### Study area and data collection

Red deer living in the North Block of the Isle of Rum, Scotland (57°03ʹN, 06°21ʹW), have been individually monitored since 1971 and regularly censused since 1974 as part of a long-term study. More than 90% of calves born in the study area are caught within a few days of birth, are weighed and marked, and tissue samples are taken for DNA analysis. For eight months of the year, weekly censuses are carried out by field workers five times a month along one of two alternating routes around the study area, recording individual locations (to the nearest 100 m × 100 m square), habitat use and behavior. Census records begin at birth for as long as the individual remains alive and in the study area. Females are philopatric, generally remaining in the same area throughout their lives, accompanied by recent offspring. Males emigrate out of the study area at about two years old but often return during the mating season.

According to previous studies (Gauzere et al., 2022; Huisman et al., 2016), the *~*12 km^2^ study area can be broadly split into six spatial regions defined by their easting (E) and northing (N) (IM, LA, MG, NG, SG and SI; see Figure 1 and Supplementary Table 1 for full names and precise co-ordinates of the spatial regions). Spatial region categorization is based on female home ranges, as most exhibit philopatric behavior (Clutton-Brock et al., 1982), and is further refined by grazing quality and population density variation (See Supplementary Figure 1). For each genotyped calf, the location was determined using census records of the mother from the beginning of May of the year the individual was born to the end of April the following year. The mean Northing and Easting coordinates of the mothers from census records over this 12-month period were either used directly in analyses or to assign spatial regions of the individual using the boundaries in Supplementary Table 1. The mean standard deviation of an individual’s northing and easting coordinates over this period were ~430 m and ~260 m, respectively, indicating that individuals exhibit relatively limited movement.

### Genomic inbreeding coefficients

Currently, there are *>*3,000 individuals with detailed life history and behavioral information that have been genotyped at 39,587 polymorphic SNPs on the 50K Cervine Illumina BeadChip (Brauning et al., 2015; Huisman et al., 2016). Individual inbreeding coefficients (*F*_ROH_) were calculated using 37,396, quality controlled autosomal SNPs of known position relative to the red deer genome assembly version mCerEla1.1 (Pemberton et al., 2021) for 3,397 genotyped individuals born between 1958 and 2023 (although majority of individuals are post 1980) using the proportion of autosomal Mb in runs of homozygosity (ROH) (McQuillan et al., 2008); see Hewett et al. (2024) for further details on quality control steps. ROH was called using *PLINK* v2.0 (Purcell et al., 2007) with the following parameters tailored to our dataset and SNP density: 2.5 Mbp minimum length and 40 SNPs minimum in an ROH; a minimum density of 1 SNP per 70 kb; 4 missing SNPs and 0 heterozygous SNPs allowed in a 35 SNP window; and a minor allele frequency threshold of 0.01. These conservative parameters allow for minimal false positives for ROH calling and, given our population and genotyping method, is likely a very accurate estimation of the true IBD (Hewett et al., 2023; Lavanchy & Goudet, 2023).

### Statistical modeling of fitness-relates traits and inbreeding coefficients

Before modeling spatial variation and ID × E, we first constructed “base” generalized linear mixed models for *F*_ROH_, birth weight and juvenile survival to identify fixed and random effects associated with variation. All base models were determined and modeled using the R (v4.2.3) package glmmTMB v1.1.7 (Brooks et al., 2017).

#### F_ROH_

We fitted *F*_ROH_ as a normally distributed response variable, square root transformed. This model included an individual's birth year as a fixed effect to account for temporal variation, fitted as a continuous number from the start of records (e.g., 1973 as 0, 1974 as 1, etc.). Model refinement shows this was the best-fitting model as *F*_ROH_ is steadily decreasing over time, although this is not significant. Mother identity (obtained through a detailed pedigree available for this population) was fitted as a random effect. The average *F*_ROH_ for all genotyped individuals with census data was 0.066 ± 0.028.

#### Birth weight

The weight of the calf at the time of capture using a Gaussian distribution was used as a proxy for birth weight (kg). The base model included the following fixed effects: The age of the calf (in hours) at the time of capture; sex; day of the year the calf was born as a continuous number from the 30th of April; mother’s age and mother’s age^2^; and the mother’s reproductive status (Levels: “True yeld”—did not give birth the previous year, “Summer yeld”—gave birth the previous year but the calf died over the summer, “Winter yeld”—gave birth the previous year but the calf died over the winter, “Milk”—gave birth the previous year and the calf survived the winter, “Naïve”—first-time breeder). See Supplementary Table 2 for base model estimated effects. Individual birth years and the mother’s identity were treated as categorical random effects. The average capture weight for all individuals used in this analysis was 7.07kg ± 1.5.

#### Juvenile survival

Juvenile survival was considered as a binary response variable, modeled using a binomial distribution with a logit link function. An individual was considered to have survived the juvenile stage if it survived until May 1^st^, 2 years after the year of birth. In this analysis, we only used individuals born in or before 2022, as this ensures all individuals have completed their juvenile survival period. For most individuals, an accurate death date is known because the corpse is found, or because they disappear from the censuses when in poor condition. A few individuals who died as a result of non-natural causes were removed from the dataset. Juveniles that were shot due to regular culling practices outside the study area were also removed from the dataset. Individuals who were shot as adults outside the study area were recorded as surviving the juvenile period. In our dataset, 57.4% of individuals are recorded to have survived the juvenile survival period. The base model included the following fixed effects: sex; the day of the year the calf was born as a continuous number from the 30th of April; mother’s reproductive status; mother’s age and mother’s age^2^, see Supplementary Table 3 for base model estimated effects. Individual birth years and the mother’s identity were treated as categorical random effects. The model was fitted with and without calf birth weight (estimated from the calf capture weight above) as a fixed effect. Results from both models are discussed, but to maximize sample size (as not every individual is weighed) and assuming that birth weight is a component of juvenile survival, we focus on the model excluding birth weight (see Supplementary Figure 2).

### Modeling of spatial variation and ID × E

The following variables were added to the base models described in the previous section. All model structures (M1–M13) are provided in Table 1.

**Table 1.** Details of models used to investigate the spatial distribution of inbreeding coefficients (*F*_ROH_), juvenile survival and capture weight (used as a proxy for birth weight). Each of these models uses a “base” model structure with additional variables, which are outlined in the main text. The statistical approach indicates the R package used for analysis. Additional explanatory variables show the fixed and random effects added to the base model. A “×” indicates the presence of an interaction term between the two variables as well as their main effects.^1^Square-root transformed *F*_ROH_ to obtain a normal distribution when *F*_ROH_ was a response variable or an explanatory variable of interest.

Model	Response variable	Statistical approach	Additional explanatory variables	Sample size
M1	F_ROH_^1^	glmmTMB	+ region	2,788
M2		R—INLA	*None*	
M3		R—INLA	+ spatial random effect	
M4	Capture weight	glmmTMB	+ region + F_ROH_	2,502
M5		glmmTMB	+ region * F_ROH_^1^	
M6		glmmTMB	+ (Northing * F_ROH_^1^) + (Easting * F_ROH_^1^)	
M7		R—INLA	+ *F*_ROH_	
M8		R—INLA	+ F_ROH_ + spatial random effect	
M9	Juvenile survival	glmmTMB	+ region + F_ROH_	2,666
M10		glmmTMB	+ region * F_ROH_^1^	
M11		glmmTMB	+ (Northing * F_ROH_^1^) + (Easting * F_ROH_^1^)	
M12		R—INLA	+ F_ROH_	
M13		R—INLA	+ F_ROH_ + spatial random effect	

#### Spatial variation

We used two alternative approaches to investigate spatial variation in *F*_ROH_, birth weight and juvenile survival. First, we modeled each variable with the spatial region as a categorical fixed effect using the R (v4.2.3) package glmmTMB v1.1.7 (Brooks et al., 2017). Second, we accounted for and quantified spatial auto-correlation using the R-INLA package (Lindgren et al., 2011; Rue et al., 2009). This approach uses the centroid location (to the nearest 100 m × 100 m) of individuals, and accounts for the fact that some individuals range closer to each other than others (e.g. individuals in the SG range closer to those in the MG than to SI or IM), therefore accounting for spatial non-independence. We provided R-INLA with an outline of the study area which is then used to create a “mesh” by dividing the area into a number of nonoverlapping triangles. Then, individual locations are overlaid on top of this mesh and weighting factors are given based on the proximity to each triangle vertex. Using an SPDE approach (continuous domain Stochastic Partial Differential Equation), a covariance matrix of distances between neighboring locations is calculated within R-INLA, allowing for the estimation of spatial autocorrelation of the data within a generalized linear mixed model. See https://github.com/annamayh/Spatial_Inbreeding_IDxE for the R-INLA code used here and Zuur, Ieno & Saveliev (2017) for further details on R-INLA. We fitted all models with and without the spatial random effect (Table 1) and compared the deviance information criteria (DIC) to assess the importance of spatial autocorrelation in the models. Similar to the AIC used for classical model selection, a reduction in DIC indicated that a model was a better fit with the inclusion of the spatial random effect, and suggests that the data are spatially autocorrelated (Zuur et al., 2017).

#### Inbreeding depression-by-environment interactions (ID × E)

To investigate ID *×* E, we fitted an interaction between spatial region as a categorical fixed effect and square root transformed *F*_ROH_ with birth weight, (M5) or juvenile survival (M9) as the response variable. We additionally fitted an interaction between square root transformed *F*_ROH_ and individual northing and easting (M6 and M11). These models were implemented in glmmTMB, as it is not currently possible to implement them in R-INLA using an interaction with SPDE.

#### Trends, predictions, and model refinements

Estimated marginal means of linear trends (M5 and M10) and pairwise comparisons between regions (M1, M4, and M8) with adjusted *p*-values were estimated using the R package emmeans v 1.10.3. Response predictions from all models run in glmmTMB used for plotting purposes were estimated using the R package ggeffects v 1.2.2 (Ludecke, 2018) adjusted for the remaining fixed effects (M1, M4, M5, M8, and M9). We also fitted a genomic relatedness matrix in models M4 and M9 to investigate the effect of accounting for population structure. There was no difference in the results, presumably because much of the population structure is captured in the spatial components of the model. Therefore, we omitted it from the reported analyses to aid computing time.

## Results

### Spatial variation of inbreeding coefficients

**Figure 2.**
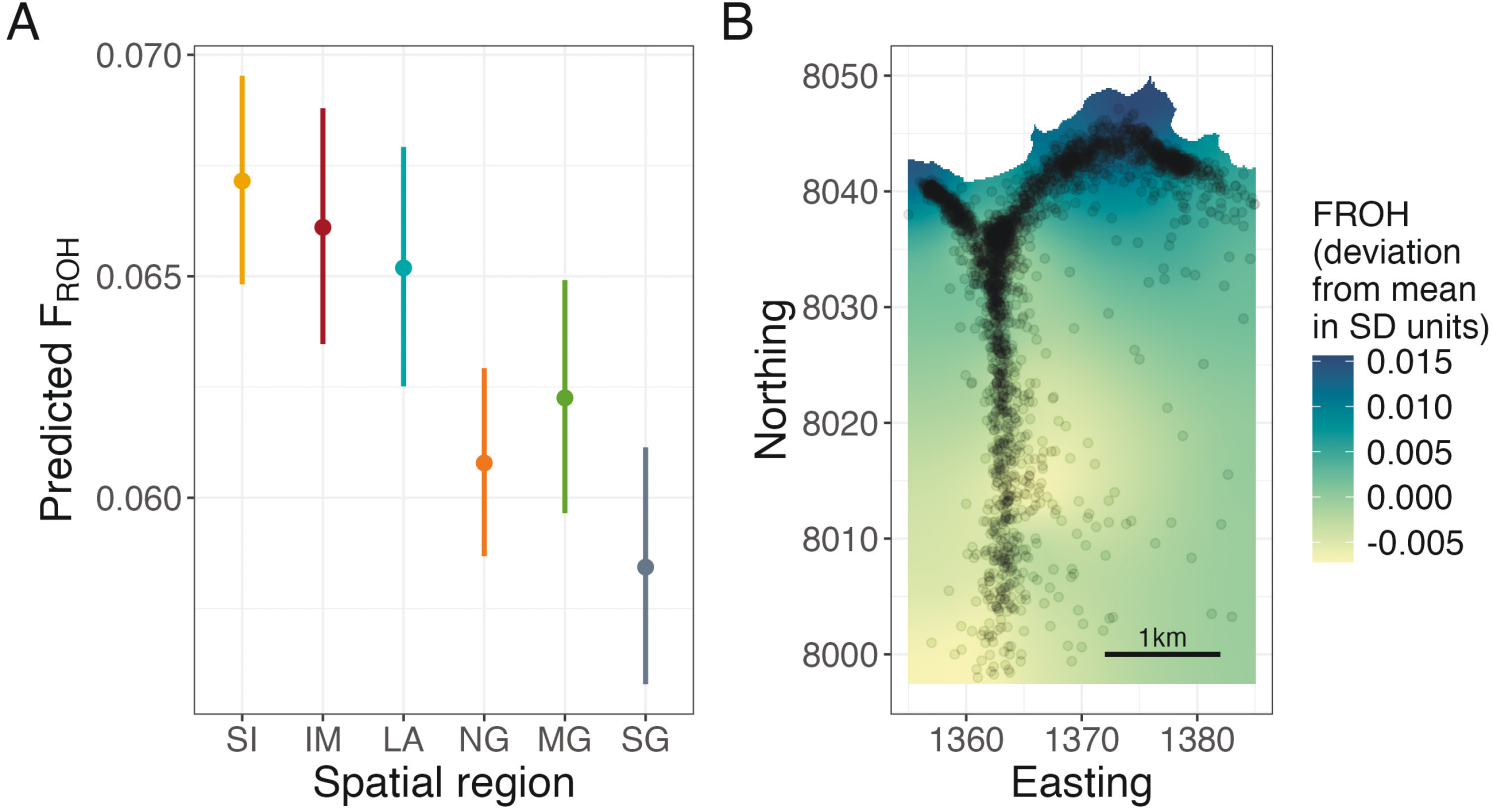
Spatial variation of inbreeding coefficients, using region as a categorical fixed effect, (A) or as a spatial random effect in R-INLA, (B). Panel (A) shows the mean predicted *F*_ROH_ per spatial region and 95% prediction intervals (nonoverlapping prediction intervals indicate significant differences between regions). Spatial region codes are provided in Figure 1. Panel (B) indicates the spatial distribution of *F*_ROH_ estimated using R-INLA with darker blue showing higher *F*_ROH_ values and lighter yellow/green lower *F*_ROH_ values, expressed as a deviation from the population mean in standard deviation (SD) units. Points indicate individual red deer positions as in Figure 1. See Supplementary Table 6 for direct model estimates.

Inbreeding coefficients differed between spatial regions (likelihood ratio test comparing models with and without region as an explanatory variable, 5 df, *p*-value: 2.97 × 10^–6^). SI and IM had higher *F*_ROH_ values on average with a mean *F*_ROH_ of 0.0698 and 0.0687 respectively, compared to NG, MG, and SG mean *F*_ROH_ values of 0.0638, 0.0650, and 0.0605, respectively. Both SI and IM had significantly higher levels of inbreeding than either the NG or SG (Supplementary Table 4, *p*-values of pairwise comparisons *<*0.05, after Tukey adjustment for multiple testing, Cohen’s *D* for SI–NG: 0.25, SI–SG: 0.37, IM–NG: 0.37, and IM–SG: 0.35). Figure 2A shows predicted mean *F*_ROH_ values across the six spatial regions and their 95% prediction intervals. In addition, the inclusion of a spatial random effect in R-INLA slightly improved the fit of the model (∆ DIC: –18.0, Supplementary Table 5) suggesting that inbreeding coefficients were partly spatially structured. There was a north–south gradient of inbreeding coefficients, with the highest *F*_ROH_ values found in the northeast of the study area (Figure 2B), corresponding to spatial region treated as a fixed effect.

### Spatial variation of fitness-related traits

**Figure 3.**
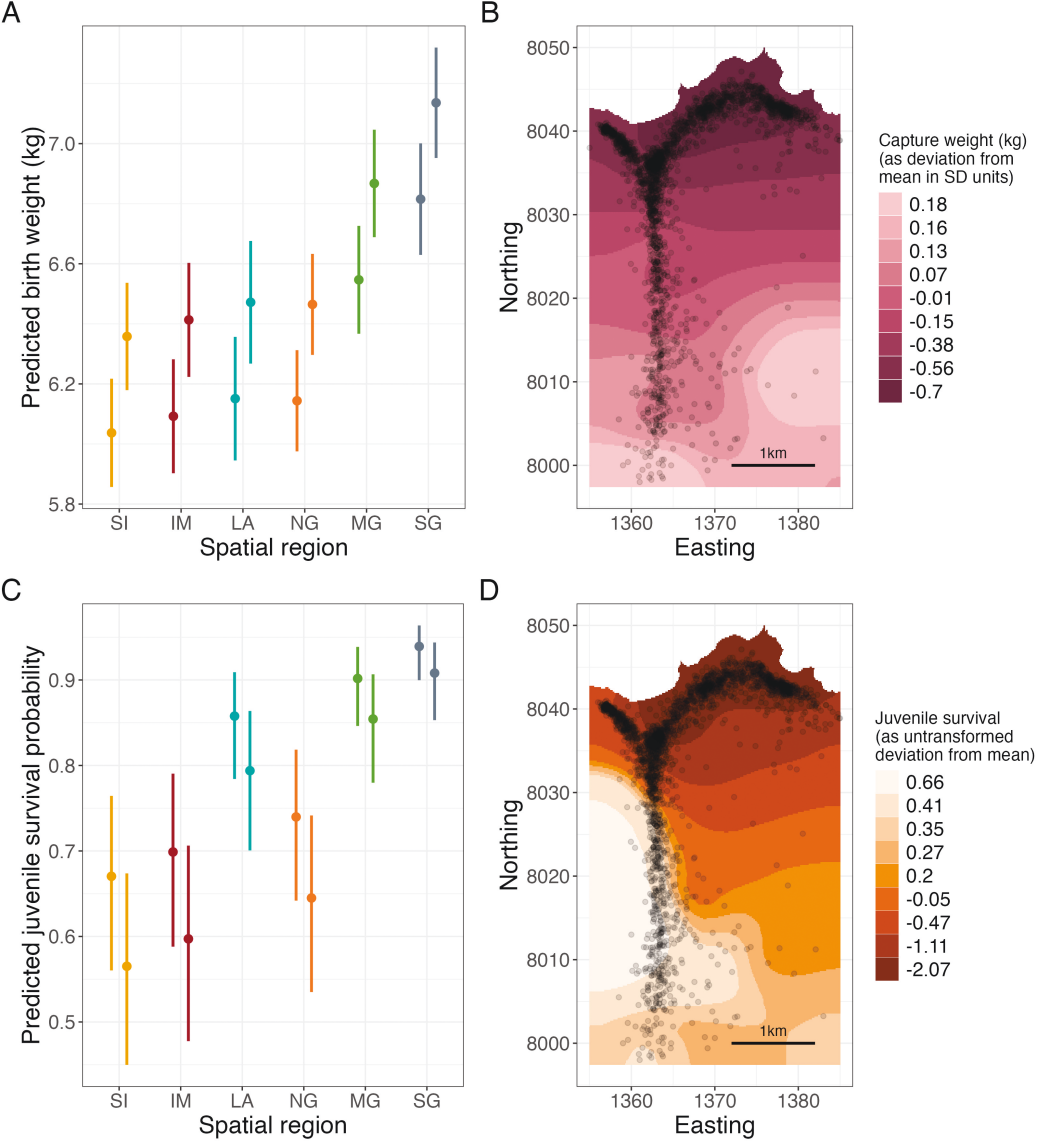
Spatial variation of fitness-related traits: Birth weight (top) and juvenile survival (bottom), using the region as a categorical fixed effect (left) or as a spatial random effect in R-INLA (right). For Panels (A and C), points show the mean predicted birth weight or juvenile survival probability per spatial region with 95% prediction intervals (nonoverlapping prediction intervals indicate significant differences between regions). Females are shown as the leftmost point within a region and males on the right. Predictions are adjusted for other fixed effects in the model and are relative to a maternal age of 8 years, the calf being born on 4th June, the *F*_ROH_ being 0.06 and the mother giving birth the previous year and the calf surviving the winter. Spatial region codes are provided in Figure 1. Panels (B and D) show the continuous spatial distribution of the traits estimated using R-INLA, with darker colors indicating lower birth weights (B) and lower juvenile survival rates (D), expressed as a deviation from the population mean (in standard deviation (SD) units in (B)). Points indicate individual red deer positions as in Figure 1. See Supplementary Tables 11 and 12 for direct model estimates.

Birth weight showed evidence for spatial variation Figure 3A and B, (likelihood ratio test comparing models with and without region as an explanatory variable, 5 df, *p*-value: 9.24 × 10^–15^). Using the categorical determinant of the region, we show SG in the south of the study area has significantly higher birth weights than all regions (Tukey method adjusted *p*-values of pairwise comparisons: *<*0.05, Supplementary Table 7). These regional differences were also consistent independent of the inbreeding level (Supplementary Table 8). After adjusting for other fixed effects in the model (see Figure 3 legend for details), the predicted birth weight of a calf in SG was 6.8 kg (95% Prediction Intervals (PIs): 6.6–7.0 kg) and 7.1 kg (95% PIs: 7.0–7.3 kg) for a female and male, respectively. In contrast, in SI a comparable calf was predicted to weigh 6.0 kg (PIs: 5.9–6.2 kg) and 6.4 kg (PIs: 6.2–6.5 kg) for a female and male, respectively, but predictions overlapped with IM, NG, and LA(Figure 3A). Inclusion of a spatial random effect in the birth weight model in R-INLA greatly improved the model fit (∆DIC: –306.5, Supplementary Table 5), supporting the evidence that birth weight differs between regions of the study area. There was a clear north–south gradient in the spatial distribution of birth weight, shown in Figure 3B, with the north of the study area having lower birth weights than the south.

Juvenile survival also differed between categorical spatial regions, see Figure 3C (likelihood ratio test comparing models with and without region as an explanatory variable, 5 df, *p*-value: <2.2 × 10^–16^), and remained independent from the level of inbreeding (Supplementary Table 9). SG, MG, and LA had higher juvenile survival probabilities than SI, IM, and NG (Tukey method adjusted *p*-values of pairwise comparisons: *<*0.01, Supplementary Table 10). For example, the predicted juvenile survival probability of a female calf born in the SG (after adjusting for other fixed effects in the model, see Figure 3 legend for details) was 94% (PIs: 90%–96%) but just 67% (PIs: 56%–76%) in SI. The inclusion of the spatial random effect also improved the fit of the model in R-INLA (∆DIC: –106.0, Supplementary Table 5). As with birth weight, there was a north–south gradient with lower survival rates in the north of the study area, including SI, IM, and NG, reinforcing the findings between regions, see Figure 3D. These spatially distributed patterns were consistent whether birth weight was fitted in the survival model or not (Supplementary Figure 2).

### Inbreeding depression-by-environment interactions (ID × E)

**Figure 4.**
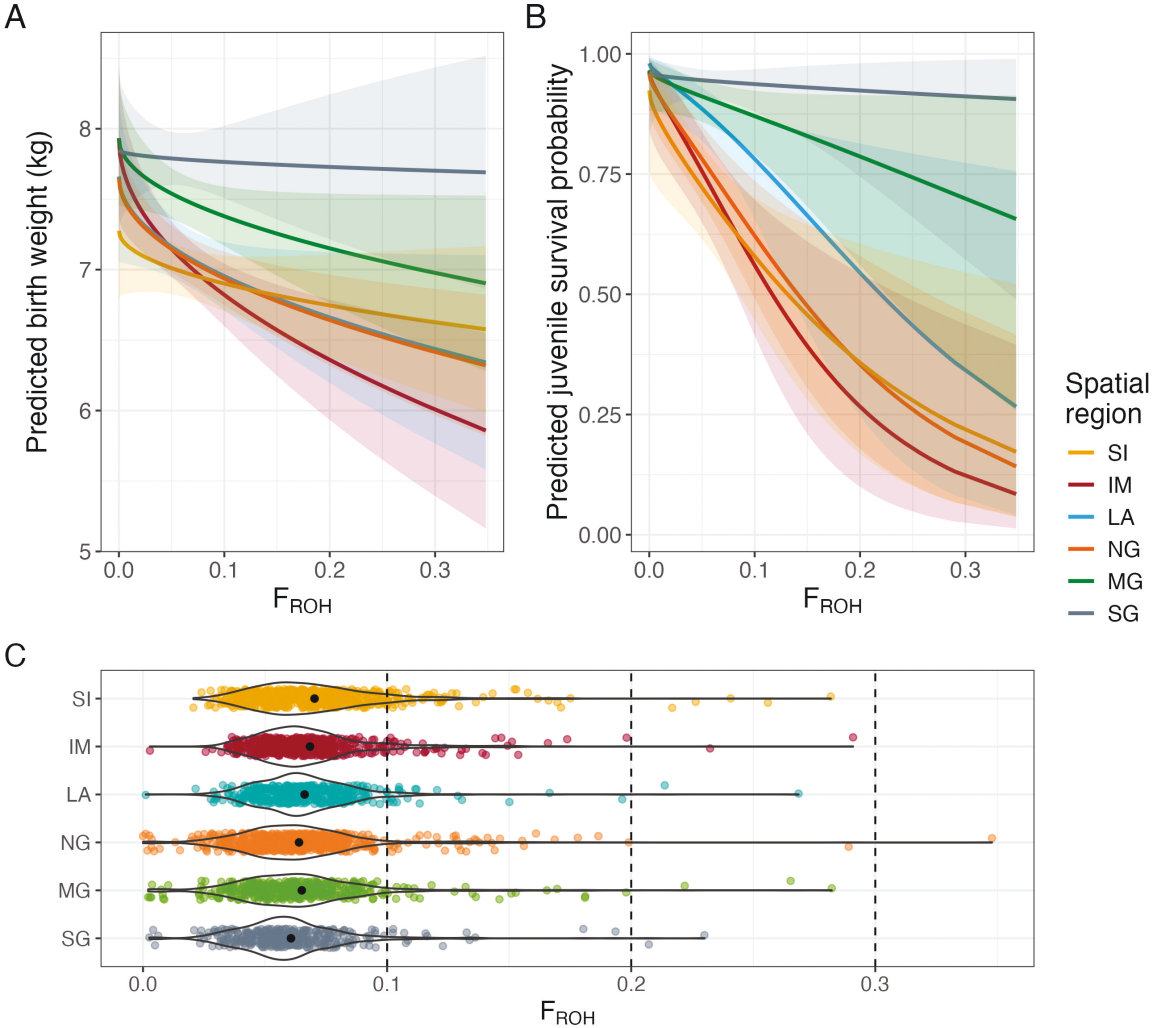
Predicted birth weight, (A) and juvenile survival probability, (B) for increasing *F*_ROH_ when an interaction is fitted with spatial region. Spatial region codes are provided in Figure 1. Mean predictions are shown as the solid line with 95% prediction intervals shown in shading of the same color. Violin plots with mean *F*_ROH_ per region, (C) and colored raw data points overlayed show the distribution of *F*_ROH_ values within each spatial region adding context to the large prediction intervals seen in (A and B) when *F*_ROH_  *>*0.2. See Supplementary Tables 16 and 18 for raw model estimates.

Models 4 and 9, fitting *F*_ROH_, show a consistent negative relationship between inbreeding coefficients and either birth weight or juvenile survival (Supplementary Tables 11 and 12), as already known from previous studies (Hewett et al., 2024; Huisman et al., 2016; Walling et al., 2011). However, some spatial regions show a stronger association than others (Figure 4 and Supplementary Tables 13 and 14). In birth weight, three of the six regions—IM, NG, and LA—show a significant decline with increased inbreeding coefficients, indicating inbreeding depression, Figure 4A and Supplementary Table 13. In contrast, in MG, SG and SI, the upper confidence level of the slope estimates overlaps with 0, indicating that there is either no relationship, or we do not have the power to detect the relationship within these regions. Consequently, a comparison of these linear trends between regions shows that none are significantly different from the other (Supplementary Table 15 and prediction intervals in Figure 4A).

We detect inbreeding depression in juvenile survival in all regions except for the SG and MG, where the upper confidence level overlaps with 0 (Supplementary Table 14). Again, this could be because inbreeding depression is not present in these regions, or we do not have the statistical power to detect it. When IM is used as the model reference level (i.e., the intercept), the difference between IM and SG is close to significant (*p*-value 0.069, Supplementary Table 16). However, after post hoc accounting for multiple tests, this is no longer the case (*p*-value 0.45), although this may be overcautious. For the other pairwise comparisons of this relationship, none are significantly different between regions (see Supplementary Table 17). Given the distribution of inbreeding coefficients within regions (shown in Figure 4C), the lack of statistical significance is perhaps not surprising. As can be seen in the wider prediction intervals in Figure 4A and B, we lack power at high *F*_ROH_, particularly when *F*_ROH_  *>* 0.2, which leads to less confidence in the estimated trends within and between regions.

Collapsing regional differences by using the northing and eastings of individuals and fitting an interaction with the inbreeding coefficient, we find a significant association of the interaction between northing and *F*_ROH_ on juvenile survival (*p*-value: 0.02), Table 2, and Supplementary Figure 3. In birth weight, there is no such effect and, in both cases, there is no association with easting and the interaction with *F*_ROH_, Table 2. Taking these results as a whole, there is evidence for ID × E when statistical power is heightened. Simplifying differences between spatial regions into a continuous variable (northing) likely still captures most of the environmental differences demonstrated between regions as well as heightening our power to detect ID × E as we use fewer degrees of freedom (df).

**Table 2.** Estimated effects (β) and standard error (SE) of inbreeding coefficient (*F*_ROH_), environmental variation (northing or easting), and an interaction term, on either capture weight (kg) or juvenile survival (on the logit scale).

	Variable	β	SE	*p*-Value
Capture weight (M6)	Northing^b^	–0.724	1.05	0.4913
	Easting^b^	–1.93	1.77	0.2756
	*F*_ROH_ ^a^	478	309	0.1214
	Northing^b^: *F*_ROH_^a^	–7.14	4.14	0.0843
	Easting^b^: *F*_ROH_^a^	6.87	6.60	0.2979
Juvenile survival (M11)	Northing^b^	1.03	3.09	0.7382
	Easting^b^	–4.56	480	0.3417
	*F*_ROH_ ^a^	2.20 × 10^3^	926	**0.0178**
	Northing^b^: *F*_ROH_^a^	–28.7	12.3	**0.0198**
	Easting^b^: *F*_ROH_^a^	7.40	18.5	0.6891

Significant effects (*p* < 0.05) are indicated in bold.

A “”: indicates the interaction term between the two variables.

^a^Square root transformed *F*_ROH_.

^b^Scaled as two-digit numbers to aid model mixing.

## Discussion

Our main aim was to investigate spatially dependent inbreeding depression (ID × E) in a wild population of red deer. We found that the northern regions of the study area had lower juvenile survival rates and birth weights compared to the southern regions (Figure 3), as shown in previous studies on this population (Coulson et al., 1997; Guinness et al., 1978a, 1978b). We further showed that these traits have a clear north–south gradient using individual locations rather than categorical regions, confirming that regions in the north are harsher environments to reside in, explained by variation in grazing quality and deer density (Coulson et al., 1997; Iason et al., 1986) and resulting in a lower overall fitness. The north of the study area also had slightly higher individual inbreeding coefficients, consistent between regional and individual location analyses, Figure 2. Our study is not the first to show that subpopulations of a wild species have different levels of inbreeding (Khan et al., 2021; Larison et al., 2021), but to our knowledge, it is the first showing inbreeding coefficients differing across continuous space at fine spatial scale. Wild subpopulations are typically spread over a wide geographical range with limited or no connections, which can lead to more inbreeding in smaller subpopulations (Khan et al., 2021). In our population, however, spatial regions directly border each other, and individuals are easily able to range between regions.

Population density and lifetime dynamics may explain why inbreeding coefficients differ across the study area. Due to the mating system of red deer, whereby males mate with a group of females, males in the north will likely have more females in their harems due to the higher population density in these areas (Albery et al., 2019; Coulson et al., 1997) and Figure 1). Indeed, Clutton-Brock et al. (1982) show that as the number of females increased in the study area after release from culling, so did the size of harems. In addition, female groups often consist of matrilineal relatives and are philopatric (Clutton-Brock et al., 1982). As a result, members of the same matriline frequently mate with the same male as each other (Stopher et al., 2012) as they are usually within the same harem. Therefore, if a male holds a large harem in an area of high density (such as SI or NG, Supplementary Figure 1) for successive years that contains many related females, the relatedness between individuals could increase faster than it would at lower density. In lions, which also have a polygynous mating system, it has also been found that in years of high population density inbreeding coefficients are higher (Trinkel et al., 2010). These factors, combined with the fact that many males that disperse as juveniles return to the study area to rut, so have relatively low effective dispersal distance (Huisman, Pemberton & n.d.), probably contribute to the higher inbreeding coefficients in the north of the study area.

Inbreeding depression also appears more severe in the north of the study area, particularly in SI, IM, and NG, Figure 4. In these regions, individuals with higher inbreeding coefficients were born lighter and were less likely to survive the juvenile stage than a completely outbred calf. In comparison, there was no detectable inbreeding depression in either birth weight or juvenile survival in the SG region. One explanation is that we simply do not have the power to detect inbreeding depression in SG. Lack of power is a common issue in quantitative analysis of natural populations (Kardos et al., 2016), particularly when searching for ID × E (Fox & Reed, (2011) and reviewed in Pemberton et al. (2017)). With an interaction term, the ability to infer ID × E is reliant on having substantial variation in inbreeding coefficients within variation in the environment. Close inbreeding is relatively rare in this study population (Hewett et al., 2023) and especially rare in SG (as seen by the lower inbreeding coefficients in the south in Figure 2 and 4C). Wild populations tend to have lower inbreeding coefficients than laboratory populations or those imposed through experimental design which means wild populations often have lower power to detect inbreeding depression. Szulkin & Sheldon (2007) specifically highlight this issue in the context of detecting ID × E. In our study, the combination of lower *F*_ROH_ and lower sample sizes in the SG (see Figure 1 for sample sizes per region) increases the uncertainty of our inbreeding depression slope in this region, which leads to lower overall confidence in ID × E. Importantly, only in cases where a reliable inbreeding depression slope can be determined for all environments, may the null hypothesis be accepted or rejected. For instance, Niskanen et al. (2020) concluded there was no evidence for ID × E in island house sparrow populations because a reliable and consistent effect of inbreeding was detected across the islands.

To increase the power of our analysis we collapsed regional differences into a continuous variable: northing and/or easting. Although this is a simplification and may disregard regional effects (e.g., LA has similar northing to SI and IM but different survival probabilities), it also reduces the number of df used to investigate ID × E and still largely captures the environmental variation. In this case, we show that individual northing is associated with the severity of inbreeding depression in survival, with more severe inbreeding depression apparent the further north an individual is born. Together, these results suggest that the strength of inbreeding depression does vary between spatial environments, indicating the presence of ID × E. Previous studies in wild populations also show traits heavily impacted by environmental factors—much the same as the effects we see of our spatial regions—can exhibit ID × E, such as hatching success (Marr et al., 2006), growth (de Boer et al., 2015; Nielsen et al., 2012), parasite burden (Coltman et al., 1999), and survival (Keller et al., 2002).

Overall, we have demonstrated spatial variation in fitness, inbreeding coefficients and inbreeding depression (ID × E) in our study population. In addition, we highlight issues of power when detecting ID × E in wild populations, where the ability to infer inbreeding depression may also be affected by the intensity of inbreeding. ID × E is undoubtedly a genuine issue that has been demonstrated multiple times in experimental systems (Armbruster & Reed, 2005; Fox & Reed, 2011) and could have extremely detrimental effects on wild populations. However, reliably detecting this interaction in situ presents considerable challenges. We suggest that future studies focusing on ID × E in natural populations should first assess the temporal or spatial variation in inbreeding coefficients to determine the power to detect inbreeding depression in different environments. Failure to do so may inflate the importance of the results and lead to an incorrect conclusion of ID × E when none exists.

## Supplementary material

Supplementary material is available online at *Evolution Letters*.

qrae073_suppl_Supplementary_Figures_S1-S3_Tables_S1-S18

## Data and code availability statement

The dataset used in this paper is published on FigShare, DOI: 10.6084/m9.figshare.28008956. All R code used is available at: https://github.com/annamayh/Spatial_Inbreeding_IDxE
