# Supplementary material for: Fine-scale spatial variation in fitness, inbreeding, and inbreeding depression in a wild ungulate
Source: Evol Lett. 2025 Jan 8;9(2):292–301. doi: 10.1093/evlett/qrae073 (PMC11968190; doi:10.1093/evlett/qrae073)
Supplement: qrae073_suppl_Supplementary_Figures_S1-S3_Tables_S1-S18 [file qrae073_suppl_supplementary_figures_s1-s3_tables_s1-s18.pdf]

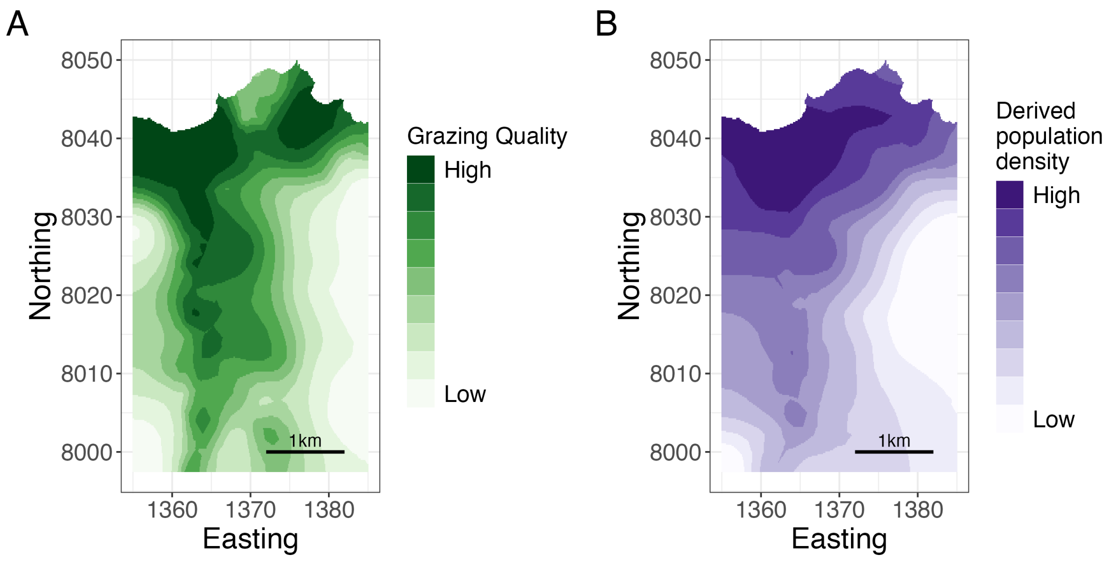

Supplementary Figure 1 – Spatial distribution of grazing quality (A) and population density (B) across the study area, estimated from R-INLA. Individual grazing quality was calculated as the proportion of time a calf's mother was sighted grazing on high-quality vegetation (defined as G1 short greens and G2 longer greens) in the calf's first year of life, where a grazing quality of 1 indicates an individual is always observed grazing on high quality vegetation and 0 is an individual never seen grazing on high quality vegetation, see Albery *et al* (2022) for further details. Individual local population densities were derived using AdeHabitatHR using the individual location centroids to calculate the spatial density of centroids, i.e. relative number of individuals per km^2, see Albery *et al* (2021) and Albery *et al* (2022) for details. For each individual, we used the derived population density of the mother during the calf's birth year.

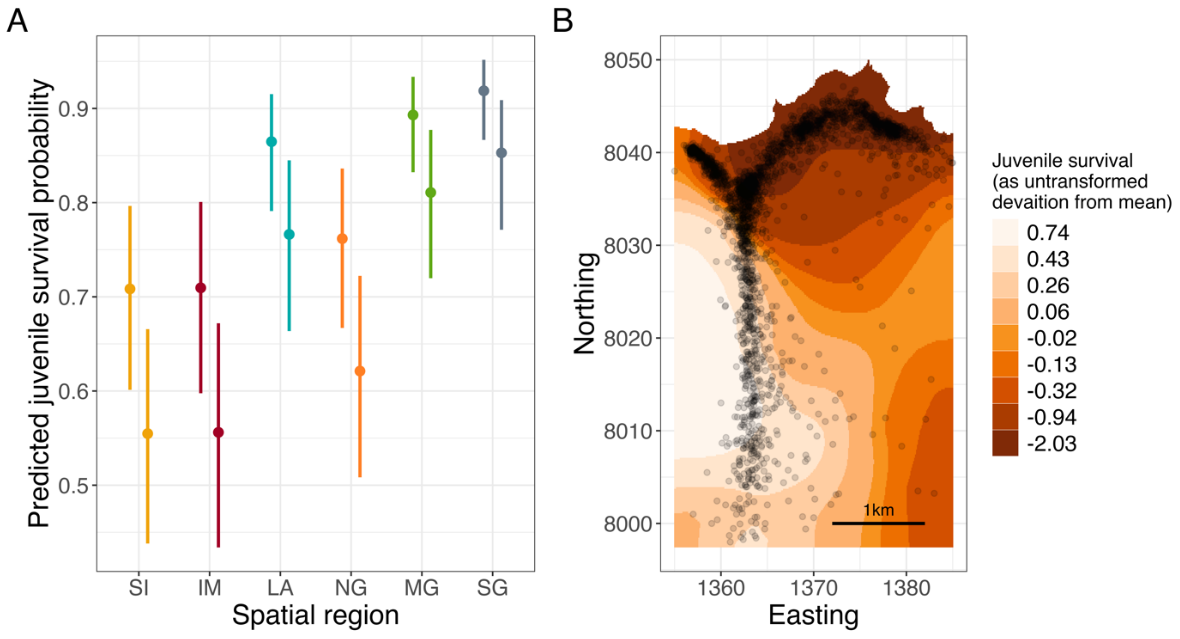

Supplementary Figure 2 – Repeat of Figure 3C and D in the main text, with the inclusion of predicted birth weight in the model of juvenile survival. Sample size: 2,413.

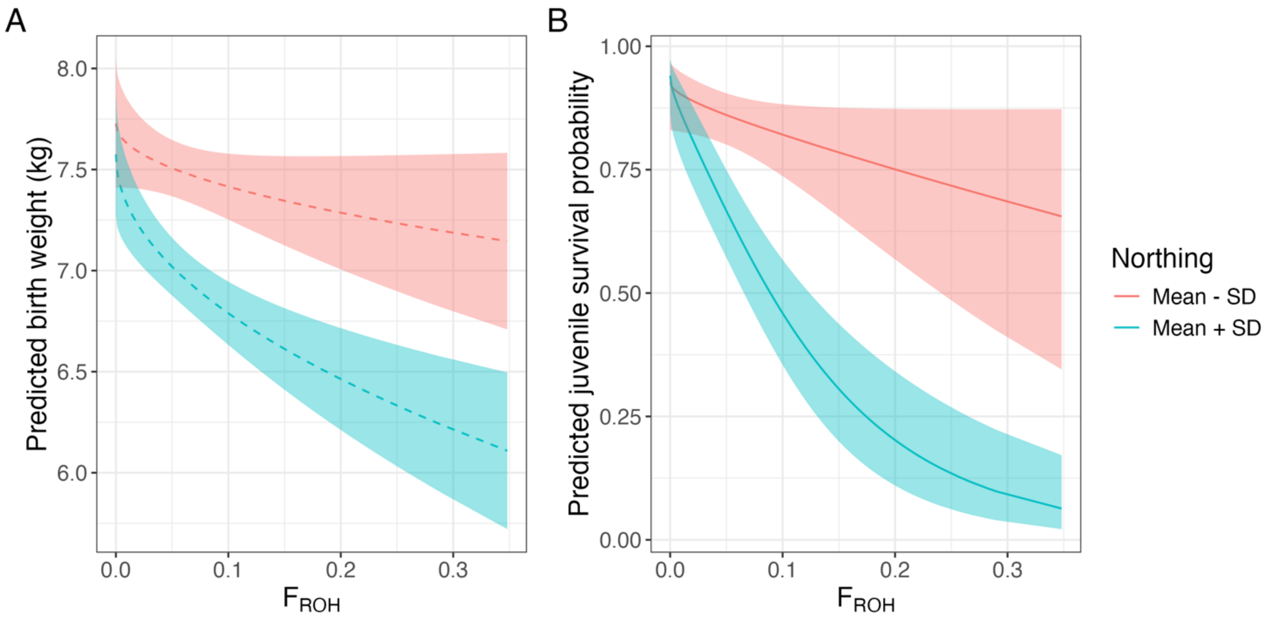

Supplementary Figure 3 – Predicted birth weight (A, Model 6), and juvenile survival probability (B, Model 11), for increased inbreeding coefficients with a northing equal to the mean ± the standard deviation (SD). Mean minus SD in pink is for a northing of 8023 and mean plus SD in blue is for a northing of 8044. A significant interaction between F_{ROH} and individual northing is indicated by a solid line whereas a dashed line indicates that the interaction was not significant in the model.

Supplementary Table 1: Isle of rum red deer study area spatial region definitions. Regions are based on female home ranges and defined using northings (N) and eastings (E). Calf regions are assigned using the average yearly N and E of the mother during the first year of the calf's life. Previous categorisations prior to Huisman *et al.* (2016) merged LA and NG and SI and IM, however, following an assessment of the grazing quality, these two regions were split into four, to better represent the different environmental conditions.

Spatial region	Easting (E)	Northing (N)
**Shamhnan insir (SI)**	=>1373 – 1385	>8019
**Laundry greens (LA)**	1355 – < 1361	>8019
**North glen (NG)**	1361 – < 1366	>8033
**Mid-glen (MG)**	1361 – <1366	8019 – 8033
**South-glen (SG)**	<1355 – >1385	< 8019 – 7997.5
**Intermediate area (IM)**	1366 - 1373	>8019

Supplementary Table 2: Base model estimated effects on capture weight (used as a proxy for birth weight). Fixed effects in order they appear in the output include: sex of the calf; age in hours; mothers reproductive status (Levels: 'True yeld' - did not give birth the previous year, 'Summer yeld' - gave birth the previous year but the calf died over the summer, 'Winter yeld' - gave birth the previous year but the calf died over the winter, 'Milk' - gave birth the previous year and the calf survived the winter, 'Naïve' - first-time breeder); mothers age and mothers age squared; the day of the year the calf was born as a continuous number from the 30th of April.

	Estimate	Std. Error	z value	p-value	significance
**(Intercept)**	3.91294	0.204335	19.15	2.00E-16	***
**Sex2**	0.3281	0.036684	8.94	2.00E-16	***
**AgeHrs**	0.016067	0.000391	41.09	2.00E-16	***
**MotherStatusNaïve**	0.036954	0.081355	0.45	0.6497	
**MotherStatusSummerYeld**	0.60257	0.069526	8.67	2.00E-16	***
**MotherStatusTrueYeld**	0.55076	0.053083	10.38	2.00E-16	***
**MotherStatusWinterYeld**	-0.20815	0.071495	-2.91	0.0036	**
**mum_age**	0.440389	0.036968	11.91	2.00E-16	***
**mum_age_sq**	-0.024135	0.001846	-13.08	2.00E-16	***
**Day_seq**	0.011122	0.001554	7.16	8.27E-13	***

Supplementary Table 3: Base model estimated effects on juvenile survival. Fixed effects in order they appear in the output include: sex of the calf; mothers reproductive status (Levels: 'True yeld' - did not give birth the previous year, 'Summer yeld' - gave birth the previous year but the calf died over the summer, 'Winter yeld' - gave birth the previous year but the calf died over the winter, 'Milk' - gave birth the previous year and the calf survived the winter, 'Naïve' - first-time breeder); mothers age and mothers age squared; the day of the year the calf was born as a continuous number from the 30th of April.

	Estimate	Std. Error	z value	p-value	significance
(Intercept)	1.383911	0.564097	2.453	0.01415	*
Sex2	-0.426437	0.102478	-4.161	3.17E-05	***
MotherStatusNaïve	-0.586488	0.221903	-2.643	0.00822	**
MotherStatusSummerYeld	-0.026192	0.188611	-0.139	0.88955	
MotherStatusTrueYeld	-0.008945	0.143432	-0.062	0.95027	
MotherStatusWinterYeld	-0.519539	0.200829	-2.587	0.00968	**
mum_age	0.297446	0.105809	2.811	0.00494	**
mum_age_sq	-0.021759	0.005374	-4.049	5.14E-05	***
Day_seq	-0.026711	0.00384	-6.955	3.52E-12	***

Supplementary Table 4: Contrasts between estimated marginal means of the 6 categorical regions using model estimates from model (M1), where F_{ROH} is the response variable. P-values are adjusted using the tukey method. Calculated using the emmeans R package.

Contrast	estimate	SE	df	t.ratio	p-value
IM-LA	0.00179	0.00379	2779	0.471	0.9971
IM-MG	0.0076	0.00378	2779	2.011	0.3364
IM-NG	0.01056	0.0034	2779	3.104	0.0237
IM-SG	0.01538	0.00387	2779	3.975	0.001
IM-SI	-0.00203	0.00349	2779	-0.582	0.9922
LA-MG	0.00581	0.0038	2779	1.528	0.6463
LA-NG	0.00878	0.00348	2779	2.524	0.1174
LA-SG	0.01359	0.00391	2779	3.479	0.0068
LA-SI	-0.00381	0.00358	2779	-1.067	0.8945
MG-NG	0.00296	0.00344	2779	0.862	0.9554
MG-SG	0.00778	0.00383	2779	2.028	0.3265
MG-SI	-0.00963	0.00355	2779	-2.709	0.0738
NG-SG	0.00481	0.00358	2779	1.346	0.759
NG-SI	-0.01259	0.00319	2779	-3.949	0.0011
SG-SI	-0.01741	0.00365	2779	-4.763	<.0001

Supplementary Table 5: Comparison of Deviance Information Criteria (DIC) for models run in R-INLA with and without the inclusion of a spatial random effect (SPDE). Lower DIC indicates the model is better fitted, regardless of sign.

Response variable	Model	DIC	ΔDIC
F_{ROH}	M2	-8833.339	-18.968
	M3<br>(Including SPDE)	-8852.307	
Capture weight	M7	7012.598	-306.495
	M8<br>((Including SPDE)	6706.103	
Juvenile survival	M12	2954.095	-106.04
	M13<br>((Including SPDE)	2848.055	

Supplementary Table 6: Estimated effects of fixed effects on F_{ROH}, model 1 (M1). 'Year_cont' represents a continuous number of birth year from the start of records to account for temporal variation. Region is fitted as a categorial fixed effect with 6 levels using the intermediate area (IM) as the intercept level.

	Estimate	Std. Error	z value	p-value
**(Intercept)**	2.58E-01	3.50E-03	73.77	2.00E-16
**year_cont**	-4.34E-05	8.10E-05	-0.54	0.59207
**RegLA**	-1.79E-03	3.79E-03	-0.47	0.63739
**RegMG**	-7.60E-03	3.78E-03	-2.01	0.04437
**RegNG**	-1.06E-02	3.40E-03	-3.1	0.00191
**RegSG**	-1.54E-02	3.87E-03	-3.98	7.04E-05
**RegSI**	2.03E-03	3.49E-03	0.58	0.56047

Supplementary Table 7: Contrasts between estimated marginal means of the 6 categorical regions using model estimates from model M4, where capture weight is the response variable. P-values are adjusted using the tukey method. Calculated using the emmeans R package.

Contrast	estimate	SE	df	t-ratio	p-value
**IM-LA**	-0.05865	0.116	2483	-0.505	0.996
**IM-MG**	-0.45431	0.104	2483	-4.373	0.0002
**IM-NG**	-0.0516	0.0944	2483	-0.546	0.9942
**IM-SG**	-0.72288	0.109	2483	-6.641	<.0001
**IM-SI**	0.05518	0.1	2483	0.55	0.994

**LA-MG**	-0.39567	0.108	2483	-3.652	0.0036
**LA-NG**	0.00705	0.103	2483	0.068	1
**LA-SG**	-0.66424	0.114	2483	-5.817	<.0001
**LA-SI**	0.11382	0.113	2483	1.012	0.9142
**MG-NG**	0.40272	0.0879	2483	4.579	0.0001
**MG-SG**	-0.26857	0.0924	2483	-2.907	0.0428
**MG-SI**	0.50949	0.101	2483	5.027	<.0001
**NG-SG**	-0.67129	0.098	2483	-6.847	<.0001
**NG-SI**	0.10677	0.0958	2483	1.114	0.8757
**SG-SI**	0.77806	0.105	2483	7.404	<.0001

Supplementary Table 8: Base model estimated effects on capture weight (used as a proxy for birth weight) with the inclusion of spatial region as a categorical fixed effect only. Other fixed effects are as described in the base model, Supplementary Table 2. Note: This differs from model 4 (M4) in the main text to determine whether the regional differences are independent from the variation in F_{ROH} between regions.

	Estimate	Std. Error	z value	p-value	significance
**(Intercept)**	3.590104	0.2164022	16.59	2.00E-16	***
**Sex2**	0.3244148	0.036486	8.89	2.00E-16	***
**AgeHrs**	0.0160063	0.0003888	41.17	2.00E-16	***
**MotherStatusNaïve**	0.064203	0.0809008	0.79	0.4274	
**MotherStatusSummerYeld**	0.6169397	0.0692221	8.91	2.00E-16	***
**MotherStatusTrueYeld**	0.565737	0.0528226	10.71	2.00E-16	***
**MotherStatusWinterYeld**	-0.1694994	0.0712313	-2.38	0.0173	*
**mum_age**	0.4586108	0.0368299	12.45	2.00E-16	***
**mum_age_sq**	-0.0248988	0.0018379	-13.55	2.00E-16	***
**Day_seq**	0.0119059	0.0015429	7.72	1.19E-14	***
**RegLA**	0.0508606	0.1165295	0.44	0.6625	
**RegMG**	0.4542419	0.1044155	4.35	1.36E-05	***
**RegNG**	0.0616223	0.0948765	0.65	0.516	
**RegSG**	0.746658	0.1092649	6.83	8.29E-12	***
**RegSI**	-0.0709631	0.1008071	-0.7	0.4815	

Supplementary Table 9: Base model estimated effects on juvenile survival, with the inclusion of spatial region as a categorical fixed effect only. Other fixed effects are as described in the base model, Supplementary Table 4. Note: This differs from model 9 (M9) in the main text to determine whether the regional differences are independent from the variation in F_{ROH} between regions.

	Estimate	Std. Error	z value	p-value	significance
**(Intercept)**	0.069587	0.575308	0.121	0.9037	
**Sex2**	-0.423193	0.099607	-4.249	2.15E-05	***
**MotherStatusNaïve**	-0.458833	0.216702	-2.117	0.0342	*
**MotherStatusSummerYeld**	-0.052944	0.184941	-0.286	0.7747	
**MotherStatusTrueYeld**	0.047773	0.139343	0.343	0.7317	
**MotherStatusWinterYeld**	-0.461173	0.195679	-2.357	0.0184	*
**mum_age**	0.416794	0.104412	3.992	6.56E-05	***
**mum_age_sq**	-0.026694	0.005296	-5.04	4.65E-07	***
**Day_seq**	-0.024422	0.003659	-6.675	2.48E-11	***
**RegLA**	0.965051	0.205865	4.688	2.76E-06	***
**RegMG**	1.372335	0.208738	6.574	4.88E-11	***
**RegNG**	0.247898	0.179873	1.378	0.1681	
**RegSG**	1.962116	0.226499	8.663	2.00E-16	***
**RegSI**	-0.148389	0.186584	-0.795	0.4264	

Supplementary Table 10: Contrasts between estimated marginal means of the 6 categorical regions using model estimates from model 9 (M9), where juvenile survival as a binomial response variable with a logit link function is the response variable. P-values are adjusted using the tukey method. Calculated using the emmeans R package.

contrast	estimate	SE	df	z.ratio	p.value
**IM-LA**	-0.955	0.208	Inf	-4.582	0.0001
**IM-MG**	-1.375	0.211	Inf	-6.503	<.0001
**IM-NG**	-0.203	0.182	Inf	-1.116	0.875
**IM-SG**	-1.896	0.229	Inf	-8.279	<.0001
**IM-SI**	0.132	0.189	Inf	0.698	0.9822
**LA-MG**	-0.42	0.217	Inf	-1.934	0.3812
**LA-NG**	0.752	0.193	Inf	3.888	0.0014
**LA-SG**	-0.942	0.233	Inf	-4.048	0.0007
**LA-SI**	1.087	0.203	Inf	5.362	<.0001
**MG-NG**	1.172	0.193	Inf	6.061	<.0001
**MG-SG**	-0.521	0.231	Inf	-2.261	0.2102
**MG-SI**	1.507	0.204	Inf	7.404	<.0001

**NG-SG**	-1.693	0.215	Inf	-7.867	<.0001
**NG-SI**	0.335	0.173	Inf	1.938	0.379
**SG-SI**	2.028	0.224	Inf	9.057	<.0001

Supplementary Table 11: Estimated effects of fixed effects on capture weight (kg) (used as a proxy for birth weight), model 4 (M4). Other fixed effects are as described in the base model, Supplementary Table 2 with the inclusion of spatial region as a categorical fixed effect and genomic inbreeding coefficient (FROH)

	Estimate	Std. Error	z value	p-value
**(Intercept)**	3.8638632	0.221619	17.43	2.00E-16
**Sex2**	0.3208557	0.0362829	8.84	2.00E-16
**AgeHrs**	0.0159765	0.0003866	41.32	2.00E-16
**MotherStatusNaïve**	0.0696999	0.0804505	0.87	0.3863
**MotherStatusSummer**	0.6148098	0.068832	8.93E+00	2.00E-16
**MotherStatusTrue**	0.5629757	0.0525298	1.07E+01	2.00E-16
**MotherStatusWinter**	-0.1650854	0.0708082	-2.33	0.0197
**mum_age**	0.4495897	0.036663	12.26	2.00E-16
**mum_age_sq**	-0.0245184	0.001829	-13.41	2.00E-16
**Day_seq**	0.0120477	0.0015345	7.85	4.11E-15
**RegLA**	0.0586464	0.1160193	0.51	0.6132
**RegMG**	0.4543133	0.1038937	4.37	1.23E-05
**RegNG**	0.0515951	0.0944482	0.55	0.5849
**RegSG**	0.7228835	0.1088449	6.64	3.11E-11
**RegSI**	-0.0551756	0.1003848	-0.55	0.5826
**FROH**	-3.5230573	0.6764601	-5.21	1.91E-07

Supplementary Table 12: Estimated effects of fixed effects on juvenile survival, model 9 (M9), where juvenile survival is treated as a binomial response variable with a logit link function. Other fixed effects are as described in the base model, Supplementary Table 4 with the inclusion of spatial region as a categorical fixed effect and genomic inbreeding coefficient (FROH).

	Estimate	SE	z value	p-value
**(Intercept)**	1.139059	0.600004	1.898	0.057641
**Sex2**	-0.44783	0.100948	-4.436	9.15E-06
**MotherStatusNaïve**	-0.483437	0.219221	-2.205	0.027437
**MotherStatusSummer**	-0.069362	0.187067	-0.371	0.710796
**MotherStatusTrue**	0.025171	0.141044	0.178	0.858363
**MotherStatusWinter**	-0.449047	0.197243	-2.277	0.022809
**mum_age**	0.380498	0.105314	3.613	0.000303
**mum_age_sq**	-0.025156	0.005331	-4.718	2.38E-06
**Day_seq**	-0.024154	0.003681	-6.562	5.30E-11

**FROH**	-13.061037	1.977825	-6.604	4.01E-11
**RegLA**	0.954841	0.208372	4.582	4.60E-06
**RegMG**	1.375151	0.211473	6.503	7.89E-11
**RegNG**	0.203283	0.18216	1.116	0.264441
**RegSG**	1.89649	0.229062	8.279	2.00E-16
**RegSI**	-0.131901	0.189035	-0.698	0.485327

Supplementary Table 13: Estimated marginal means of linear trends within regions for model M5 - Capture weight as the response variable including an interaction between categorical spatial region and inbreeding coefficient. F_{ROH} trend is the estimated difference between having an F_{ROH} of 0 or having an F_{ROH} of 1. SE indicates the standard error of the slope. Lower and upper CL indicates the 95% lower and upper confidence levels of the slope. Overall significance of the slopes can be determined if the upper - lower CL does not overlap zero.

Reg	FROH_trans.trend	SE	df	lower.CL	upper.CL
**IM**	-3.517	1.02	2478	-5.52	-1.5102
**LA**	-2.237	1.12	2478	-4.44	-0.0339
**MG**	-1.738	0.897	2478	-3.5	0.0216
**NG**	-2.254	0.703	2478	-3.63	-0.8746
**SG**	-0.268	1.17	2478	-2.57	2.0306
**SI**	-1.185	0.873	2478	-2.9	0.5259

Supplementary Table 14: Estimated marginal means of the linear slope of inbreeding depression in juvenile survival (on the logit scale) within 6 spatial regions of the study area. F_{ROH} slope is the estimated difference between having an F_{ROH} of 0 or having an F_{ROH} of 1. SE indicates the standard error of the slope. Lower and upper CL indicates the 95% lower and upper confidence levels of the slope. Overall significance of the slopes can be determined if the upper - lower CL does not overlap zero.

Reg	FROH_trans.trend	SE	df	asymp.LCL	asymp.UCL
**IM**	-9.59	2.9	Inf	-15.26	-3.912
**LA**	-8.35	3.19	Inf	-14.6	-2.111
**MG**	-4.59	2.49	Inf	-9.47	0.286
**NG**	-8.39	2.08	Inf	-12.46	-4.323
**SG**	-1.59	3.31	Inf	-8.08	4.905
**SI**	-6.89	2.45	Inf	-11.7	-2.088

Supplementary Table 15: Contrast of estimated marginal means of linear trends between regions for model M5 - Capture weight as the response variable including an interaction between categorical spatial region and inbreeding coefficient. P-values are adjusted using the tukey method. Calculated using the emmeans R package.

contrast	estimate	SE	df	t ratio	p-value
**IM-LA**	-1.28	1.52	2478	-0.844	0.9592
**IM-MG**	-1.7789	1.36	2478	-1.311	0.7793
**IM-NG**	-1.2627	1.24	2478	-1.019	0.9117
**IM-SG**	-3.2485	1.55	2478	-2.091	0.2921
**IM-SI**	-2.3315	1.34	2478	-1.735	0.5083
**LA-MG**	-0.4989	1.44	2478	-0.348	0.9993
**LA-NG**	0.0173	1.32	2478	0.013	1
**LA-SG**	-1.9685	1.62	2478	-1.213	0.8309
**LA-SI**	-1.0515	1.42	2478	-0.74	0.9769
**MG-NG**	0.5162	1.14	2478	0.454	0.9976
**MG-SG**	-1.4696	1.48	2478	-0.99	0.9213
**MG-SI**	-0.5526	1.26	2478	-0.44	0.9979
**NG-SG**	-1.9858	1.37	2478	-1.455	0.6933
**NG-SI**	-1.0688	1.13	2478	-0.948	0.9338
**SG-SI**	0.917	1.46	2478	0.628	0.989

Supplementary Table 16: Direct model estimates from model M9 – Juvenile survival as the response variable including an interaction between categorical spatial region (Reg) and inbreeding coefficient (FROH). Here region IM (intermediate) is the reference level.

**(Intercept)**	**2.761477**	**0.936818**	**2.948**	**0.0032**
**Sex2**	-0.44869	0.100206	-4.478	7.55E-06
**MotherStatusNaïve**	-0.507278	0.217393	-2.333	0.01962
**MotherStatusSummer**	-0.084769	0.184816	-0.459	0.64647
**MotherStatusTrue**	0.002764	0.139996	0.02	0.98425
**MotherStatusWinter**	-0.461285	0.196261	-2.35	0.01876
**mum_age**	0.369096	0.103862	3.554	0.00038
**mum_age_sq**	-0.024479	0.005252	-4.661	3.15E-06
**Day_seq**	-0.024253	0.003672	-6.604	3.99E-11
**RegLA**	0.640616	1.111857	0.576	0.5645

**RegMG**	0.083399	0.990386	0.084	0.93289
**RegNG**	-0.124391	0.914812	-0.136	0.89184
**RegSG**	-0.070857	1.112753	-0.064	0.94923
**RegSI**	-0.777344	0.984824	-0.789	0.42992
**FROH_trans**	-9.586749	2.895474	-3.311	0.00093
**RegLA:FROH_trans**	1.232127	4.276403	0.288	0.77325
**RegMG:FROH_trans**	4.993538	3.804585	1.313	0.18935
**RegNG:FROH_trans**	1.196555	3.549542	0.337	0.73604
**RegSG:FROH_trans**	7.997287	4.395217	1.82	0.06883
**RegSI:FROH_trans**	2.69429	3.777951	0.713	0.47575

Supplementary Table 17: Contrast of estimated marginal means of linear trends between regions for model M9 - Juvenile survival as the response variable including an interaction between categorical spatial region and inbreeding coefficient. P-values are adjusted using the tukey method. Calculated using the emmeans R package.

contrast	estimate	SE	df	z ratio	p-value
**IM-LA**	-1.2321	4.28	Inf	-0.288	0.9997
**IM-MG**	-4.9935	3.8	Inf	-1.313	0.7783
**IM-NG**	-1.1966	3.55	Inf	-0.337	0.9994
**IM-SG**	-7.9973	4.4	Inf	-1.82	0.453
**IM-SI**	-2.6943	3.78	Inf	-0.713	0.9804
**LA-MG**	-3.7614	4.04	Inf	-0.931	0.9387
**LA-NG**	0.0356	3.78	Inf	0.009	1
**LA-SG**	-6.7652	4.59	Inf	-1.475	0.6802
**LA-SI**	-1.4622	4.01	Inf	-0.365	0.9992
**MG-NG**	3.797	3.24	Inf	1.173	0.8499
**MG-SG**	-3.0037	4.15	Inf	-0.723	0.9791
**MG-SI**	2.2992	3.51	Inf	0.655	0.9867
**NG-SG**	-6.8007	3.91	Inf	-1.738	0.5062
**NG-SI**	-1.4977	3.22	Inf	-0.465	0.9973
**SG-SI**	5.303	4.12	Inf	1.286	0.7929

Supplementary Table 18: Estimated effects of fixed effects and standard error on capture weight (used as a proxy for birth weight), model 5 (M5). Other fixed effects are as described in the base model, Supplementary Table 2 with the inclusion of an interaction between categorical spatial region (Reg) and inbreeding coefficient (FROH). Here region IM (intermediate) is the reference level.

	Estimate	SE	z value	p-value
(Intercept)	4.5160336	0.3402018	13.27	2.00E-16
Sex2	0.3229662	0.0362943	8.9	2.00E-16
AgeHrs	0.0159762	0.0003866	41.33	2.00E-16
MotherStatusNaïve	0.0711246	0.0804366	0.88	0.376571
MotherStatusSummer	0.6104799	6.88E-02	8.87E+00	2.00E-16
MotherStatusTrue	0.5600988	5.25E-02	1.07E+01	2.00E-16
MotherStatusWinter	-0.1616011	0.0707965	-2.28	0.022453
mum_age	0.4524199	0.036675	12.34	2.00E-16
mum_age_sq	-0.0246478	0.0018294	-13.47	2.00E-16
Day_seq	0.0119883	0.0015333	7.82	5.33E-15
RegLA	-0.2716617	0.4081972	-0.67	0.505721
RegMG	-0.0046757	0.3592932	-0.01	0.989617
RegNG	-0.2830229	0.328873	-0.86	0.389467
RegSG	-0.0841535	0.3979548	-0.21	0.832524
RegSI	-0.657122	0.362908	-1.81	0.070185
FROH_trans	-3.5168066	1.0232871	-3.44	0.000589
RegLA:FROH_trans	1.2800455	1.5170053	0.84	0.398782
RegMG:FROH_trans	1.7789312	1.3572712	1.31	0.18997
RegNG:FROH_trans	1.2627444	1.2391958	1.02	0.308201
RegSG:FROH_trans	3.2485174	1.5534563	2.09	0.036514
RegSI:FROH_trans	2.3315351	1.3435183	1.74	0.082671
